# Assessment of fecal steroid and thyroid hormone metabolites in eastern North Pacific gray whales

**DOI:** 10.1093/conphys/coaa110

**Published:** 2020-12-07

**Authors:** Leila S Lemos, Amy Olsen, Angela Smith, Todd E Chandler, Shawn Larson, Kathleen Hunt, Leigh G Torres

**Affiliations:** 1 Fisheries and Wildlife Department, Marine Mammal Institute, Oregon State University, 2030 SE Marine Science Dr, Newport, OR 97365, USA; 2 Conservation Programs and Partnerships, Seattle Aquarium, 1483 Alaskan Way, Seattle, WA 98101, USA; 3 Smithsonian-Mason School of Conservation, 1500 Remount Road, Front Royal, VA 22630, USA

**Keywords:** Androgens, eastern North Pacific gray whale, fecal hormone metabolites, glucocorticoids, progestins, thyroid

## Abstract

Baleen whale fecal samples have high potential for endocrine monitoring, which can be used as a non-invasive tool to identify the physiological response to disturbance events and describe population health and vital rates. In this study, we used commercial enzyme-linked immunosorbent assays to validate and quantify fecal steroid (progestins, androgens and glucocorticoids) and thyroid hormone metabolite concentrations in eastern North Pacific gray whales (*Eschrichtius robustus*) along the Oregon coast, USA, from May to October of 2016–2018. Higher mean progestin metabolite concentrations were observed in postweaning females, followed by pregnant females. Mean androgen, glucocorticoid and thyroid metabolites were higher in mature males. Progestin, glucocorticoids and thyroid fecal metabolites varied significantly by year, with positive correlations between progestin and androgen, and between glucocorticoid and thyroid metabolites. We also present two case studies of a documented injured whale and a mature male displaying reproductive competitive behavior, which provide reference points for physiologically stressed individuals and adult breeding males, respectively. Our methods and findings advance the knowledge of baleen whale physiology, can help guide future research on whale physiology and can inform population management and conservation efforts regarding minimizing the impact of anthropogenic stressors on whales.

## Teaser

An improved understanding of baleen whale hormone physiology is required to manage populations and reduce anthropogenic impacts. Gray whale fecal samples were assessed, and hormone metabolites quantified, which varied by demographic units and years. Two case studies are presented providing reference points for physiologically stressed individuals and adult breeding males.

## Introduction

Anthropogenic impacts on the environment have increased as human populations continue to grow, creating a variety of environmental pressures to many wildlife species (Wilson, 1999; Wikelski and Cooke, 2006; Acevedo-Whitehouse and Duffus, 2009; Toukhsati, 2017). These pressures can be acute or chronic stressors, stimulating varied physiological responses in wildlife, including body condition deterioration, immunosuppression, infections, cognition impairments, decrease in reproductive hormones and population fitness, increase in stress-associated hormones and mortality (Wikelski and Cooke, 2006; Acevedo-Whitehouse and Duffus, 2009; Cooke and O’Connor, 2010; Homyack, 2010). Therefore, assessment and monitoring of wildlife health in response to anthropogenic stressors is critical to identify impacts and elucidate potential causes of physiological shifts. Only once identified can these impacts/stressors may be abated.

In all vertebrates, a physiological response to a disturbance is referred to as a *stress response*, a complex physiological cascade of events that helps the individual to recover from, or cope with these stressors (Selye, 1950; Moberg, 2000; Schreck *et al.*, 2016). This cascade involves neural and hormonal reactions, eliciting the synthesis and secretion of catecholamines and glucocorticoids (GC; cortisol and related compounds) into the blood (Moberg, 2000; Touma and Palme, 2005; Romero and Wingfield, 2016; Schreck *et al.*, 2016). Catecholamines are typically too labile and short-lived to be accurately quantifiable in a wildlife research context, while blood GC concentrations are relatively easy to assess and have been widely used to evaluate acute and chronic stress responses in wildlife (Place and Kenagy, 2000; Champagne *et al.*, 2012, 2017; Fair *et al.*, 2014; Stier *et al.*, 2019).

Baleen whale physiology is probably the most poorly understood of all vertebrates (Hunt *et al.*, 2006, 2013). A number of logistic complications arise when studying the physiology of large and aquatic animals that only spend brief periods of time at the surface, are nearly impossible to capture alive and are ethically and logistically impossible to capture unharmed. Hence, collecting blood samples regularly to measure physiological state or reactions in these animals is not viable as there is no current method for blood collection from live, free-swimming mysticete whales (Amaral, 2010; Hunt *et al.*, 2013). Fortunately, innovative methods have been developed to assess the physiology of wild animals with minimal to no impact on animal stress levels (Wasser *et al.*, 2000; Wikelski and Cooke, 2006). For example, a range of other sample types can be collected from baleen whales for physiological analyses, including feces, respiratory vapor (i.e. blow) and blubber biopsy samples (Rolland *et al.*, 2005; Kellar *et al.*, 2013; Burgess *et al.*, 2016, 2017, 2018; Pallin *et al.*, 2018; Hunt *et al.*, 2019). With the exception of blow samples, which appear to mirror patterns from blood hormone concentrations (Tizzi and Accorsi, 2010; Richard *et al.*, 2017), the other matrices reflect the metabolized products (i.e. hormone metabolites; HM) of the parent hormones.

Steroid (i.e. progestins, estrogens, androgens, GC, and mineralocorticoids) and thyroid (i.e. triiodothyronine—T3—and thyroxine—T4) hormones are primarily cleared from the blood by the liver in most vertebrates, and then metabolized and excreted via feces or urine (Palme *et al.*, 1996). These hormones are excreted into the gut via the bile ducts and modified by gut bacteria (Taylor, 1971; Touma and Palme, 2005; Goymann, 2012). The lag time between the hormone release into the blood and peak hormone excretion in feces is species dependent and typically takes from 4 to 48 h in mammals, such that fecal hormone data likely reflect an integrated average of previously secreted hormones of the previous 1–2 days (Palme *et al.*, 1996; Touma and Palme, 2005; Amaral, 2010; Wasser *et al.*, 2010). Feces can provide several advantages compared to other biological samples, including replicate non-invasive sampling of individuals over multiple time periods, high detectability of HM and well-established analytical techniques (Wasser *et al.*, 2010; Hunt *et al.*, 2013; Palme, 2019). Variation introduced by intrinsic and extrinsic factors like age, sex, metabolic rate, food intake, environmental parameters, reproduction, pregnancy and lactation (Foley *et al.*, 2001; Mormède *et al.*, 2007; Goymann, 2012; Romero and Wingfield, 2016) can be minimized by (1) working with known individuals and, thus, using available information on sighting history, sex, approximate age and reproductive status, (2) collecting samples during the same life history phase in populations that largely consume the same diet (Goymann, 2012; Stetz *et al.*, 2013) and (3) analysing reproductive HM (i.e. progestins and androgens) in parallel with GCs so as to identify effects of sex and reproductive state on GCs (e.g. Hunt *et al.*, 2006; Corkeron *et al.*, 2017).

Analytical and physiological validations are crucial initial steps in hormone studies. Analytical validations verify if the assay antibody is binding well to the hormone/HM (i.e. parallelism) and if the assay can distinguish between low and high hormone/HM concentrations (i.e. accuracy or ‘matrix test’; Grotjan and Keel, 1996; Hunt *et al.*, 2013; Palme, 2019). Physiological validations determine if the measured hormone/HM accurately reflects the physiological state of the animal (Palme, 2005). To date, a series of fecal analytical validations have been conducted in mysticete whales including progestin, estrogen, androgen, GC and mineralocorticoid metabolites in North Atlantic right whales (NARWs—*Eubalaena glacialis*; Rolland *et al.*, 2005; Hunt *et al.*, 2006; Burgess *et al.*, 2017), progestin and GC metabolites in eastern North Pacific (ENP) blue whales (*Balaenoptera musculus*; Valenzuela-Molina *et al.*, 2018) and progestin, estrogen, androgen, GC and thyroid metabolites in North Atlantic humpback whales (Hunt *et al.*, 2019).

When interpreting results of HM analyses, it is especially important to differentiate baseline from stress-related HM concentrations. Baseline concentrations should reflect the normal range of HM in a healthy population, while stress-related concentrations are typically outside the normal range (usually above; Cyr and Romano, 2009; Hunt *et al.*, 2013). In order to identify abnormal HM concentrations, a firm understanding of the normal range of HM concentrations within a specific population is needed. Working with a large dataset composed of replicate samples collected over multiple years from individuals with associated information on demographic unit, reproductive and health status can assist in development of this baseline understanding needed to differentiate between normal and abnormal HM concentrations.

Fecal endocrine studies of gray whales (*Eschrichtius robustus*) to establish analytical methods, physiological patterns and utility for conservation and management have not yet been conducted. Gray whales are an evolutionarily distinct lineage of baleen whale, traditionally placed in their ownfamily *Eschrichtiidae* (Jones *et al.*, 2012). There is only one surviving species in this genus, which is now restricted to the Pacific Ocean, including the Western North Pacific population that is critically endangered (~140 individuals; Cooke *et al.*, 2013), and the ENP population that has made a remarkable recovery since depletion from whaling from 1000–2000 (Moore *et al.*, 2001) to 26 960 individuals (coefficient of variance of 0.05; Durban *et al.*, 2017).

The ENP gray whales undergo an annual migration from their southern breeding grounds in Baja California, Mexico, to their northern feeding grounds in the Bering and Chukchi seas near Alaska, USA (Calambokidis *et al.*, 2002; Sumich, 2014). However, not all gray whales perform the full migration; a group of ~200–250 individuals (Calambokidis and Perez, 2017) can be found feeding along the coasts from northern California to southeastern Alaska during the summer, forming the Pacific Coast Feeding Group (PCFG; Rice and Wolman, 1971; Calambokidis *et al.*, 2002, 2015). These whales usually remain within 10 km of the shore (Mate and Urban-Ramirez, 2003; Sumich, 2014) and thus are subject to a number of anthropogenic pressures, including fishing gear entanglement, coastal polllution, vessel/ship encounters and strikes and ambient noise (Baird *et al.*, 2002; Moore and Clarke, 2002; Jones *et al.*, 2012). The ENP was delisted from the US Endangered Species Act in 1994 (Federal Register, 1993); however, two unusual mortality events of unknown causes in 1999–2000 (Gulland *et al.*, 2005) and in2019–2020 (NOAA, 2020) have resulted in relatively high numbers of strandings of dead gray whales when compared to other years (1999–2000: 651 stranded whales; and 2019–2020: 370 stranded whales). Therefore, there is a pressing need for development of non-invasive methods to assess the stress and reproductive physiology in baleen whales generally, and in ENP gray whales specifically.

In this study, we describe variations in fecal steroid (i.e. progestins, androgens and GC) and thyroid (i.e. T3) metabolite concentrations in PCFG gray whales. The objectives of this study are to (1) provide analytical validations (precision, sensitivity, parallelism and accuracy) of immunoreactive hormone metabolites in gray whale fecal samples, (2) describe baseline concentrations of progestin, androgen, GC and thyroid metabolites, and determine whether fecal metabolite concentrations significantly vary according to demographic units, reproductive state and health status, (3) assess temporal variations in fecal HM concentrations and (4) evaluate potential correlations between the different HMs.

## Materials and methods

### Sampling methods

PCFG gray whales forage along the Oregon coast, USA, between late May and October (Calambokidis *et al.*, 2002). Therefore, we used a 5.4 m rigid-hulled inflatable boat to locate gray whales and collect individual-based data along the Oregon coast during the summer foraging period in the years of 2016, 2017 and 2018. Sample collection occurred in two different gray whale foraging regions as part of a larger research program on gray whale ecology including assessment of whale movement and behavior patterns relative to habitat and prey availability: Newport (44°38′13”N, 124°03′08”W), in central Oregon, and Port Orford (42°44′59”N, 124°29′53”W), in southern Oregon. These two regions are ~200 km apart and have the same habitat types, whale behavior and prey availability, along with inter-change of the same individual whales between regions (Sullivan and Torres, 2018; Torres *et al.*, 2018; *L. Torres unpub. data*). Two cameras (Canon EOS 7D and Canon EOS 70D) were used to obtain whale photographs of the left-hand side, right-hand side and fluke of each individual for photo-identification (photo-id). If whales were behaving naturally (i.e. no behavioral changes due to the presence of the boat) and weather conditions were appropriate (e.g. low wind, no fog), we conducted unmanned aerial system (i.e. UAS; a.k.a. drone) overflights of the whales for behavioral and body condition assessment through photogrammetry methods (methods described in Lemos *et al.*, 2020). The identity of each whale recorded in a drone video was confirmed through photo-id images.

Gray whale fecal samples were opportunistically collected when defecation events were observed from the boat or during drone flight observations. Two 300 μm nylon mesh dipnets were dragged through the plume multiple times to capture as large a fecal sample as possible. The location, date, time and photo-id images of the sampled whale were recorded at each fecal sample collection. Often a whale would defecate multiple times at the terminal dive of sequential surface series that are about 4 mins apart (L. Lemos, *pers. obs.*). These defecations were collected in the same nets to increase sample size. In these instances, all subsamples collected during the same sighting for the same whale were assigned the location, date and time information from the first sampling location. Using ambient seawater in squeeze bottles, the fecal sample material from the two nets was flushed out of the net into a single sterile plastic jar and put on ice until we returned to shore and stored in a freezer (−20°C) for later analysis.

Four additional fecal samples from dead stranded gray whales along the Oregon and Washington coasts, USA, were collected by stranding response network collaborators. These samples were analysed, and their GC concentrations were interpreted as typical endocrine profiles of physiologically stressed individuals (Appendix S1: Table S1). These fecal samples were obtained by cutting into the caudal-most aspect of the colon and collecting the fecal matter. The samples were placed into sterile whirl-paks or jars and stored in a freezer (−20°C) until analysis.

### Analysis methods

#### Whale identification and assignment to demographic units

Whale identification photographs were assessed using Adobe Bridge (version 8.0.1.282) and photo quality was ensured by only analysing images in focus, well lit and not affected by glare, angle or distance (Hammond *et al.*, 1990; Calambokidis *et al.*, 2010). We compared individual whale images to long-term gray whale photo-id catalogs (>30 years) held by the Marine Mammal Institute at Oregon State University and Cascadia Research Collective (Olympia, WA), in order to obtain information on sex (based on previous tissue genetic analysis; Lang *et al.*, 2014) and minimum age (based on date of first sighting). When sex information was not available, fecal sample genetic analyses for sex determination were performed (fecal genetic analysis method described in Lemos *et al.*, 2020).

Gray whale females have a reproductive cycle of 2 years (Rice *et al.*, 1984), with conception occurring during the southbound migration (i.e. December–January). Gestation lasts ~13 months and most births occur during the month of January. The lactation period lasts around 7 months, usually ending in August. Based on the literature regarding gray whale reproductive cycles, our photo-id and photogrammetry results (Lemos *et al.*, 2020), and fieldwork observations, each identified whale was assigned to one of the following demographic units: calf, immature male, immature female, immature of unknown sex, mature male, resting female, pregnant female, lactating female, postweaning female, mature of unknown sex and undetermined. Resting females represent mature females that were not pregnant or lactating in the analysis year. Lactating females represent relatively large whales swimming in close association with small whales (<8 m in length) that were assigned as calves (Perryman and Lynn, 2002). It was assumed that a lactating female seen later in the season unaccompanied by its calf was a postweaning female, and that the lactating female was a pregnant female in the previous year.

Sexual maturity assignment followed Zimushko (1969) and Zimushko and Ivashin (1980) determinations: females become mature between 8 and 12 years (~12 m in length) and males become mature between 6 to 8 years (~11.5 m in length). In order to attain certainty in the maturity classification, 7 years was used as the maximum age cut-off value for immature females and 12 years as the minimum value for mature females. In males, these values were 5 and 8 years, respectively. Individuals at a length range between 10 and 12 m of unknown sex or age were classified as ‘undetermined’.

Based on indications that gray whales are impregnated during the southbound migration (Rice *et al.*, 1984) we assume that all fecal samples from pregnant females were collected at around 6–9 months of gestation.

#### Injury and infection assessment

We reviewed the drone recordings (VLC Media Player software; version 2.2.8) and all photos of whales for evidence of injuries or infections. Whales with identified moderate to severe injuries or infections within 48 h from a fecal sample collected were categorized as ‘known-stressed whales’. Injuries were classified as moderate (i.e. not life threatening) when a laceration penetrated through blubber to muscle, and as severe if there was any injury to the bone or abdominal cavity.

#### Fecal sample preparation

The first step in fecal sample preparation for analysis was to drain saltwater from all samples by using unbleached coffee filters. In order to prevent salt weight from inflating apparent dried fecal mass, deionized water was then added to all samples to rinse any remaining salts from fecal particulates. The samples were then centrifuged for 10 min at 3000 rpm (i.e. 1000 RCF—relative centrifugal force [g]), and overlying water was removed by pipetting. Note that mammalian fecal hormone metabolites can include both hydrophilic metabolites and hydrophobic metabolites; it was expected that hydrophilic metabolites had already been lost to seawater upon defecation, such that additional aqueous rinse steps after collection would not further alter hormone content. Nonetheless, extracted water was saved, frozen and later tested to determine if there was any HM loss from feces into the overlying water. Samples (i.e. rinsed and centrifuged fecal particulates) were then frozen and lyophilized (i.e. freeze dried) for 72 h so as to remove all water content. Therefore, all hormone values reported in this study are per g dried fecal mass. All samples were analysed for HM concentration within 11 months of collection.

#### Hormone metabolite extraction

Processed samples were well mixed and weighed in glass tubes within a range of 0.02 and 0.2 g. A cut-off weight point of 0.02 g was used to guarantee the accuracy of the HM concentration and avoid inflated values (Millspaugh and Washburn, 2004). This cut-off point was determined after plotting sample weight (g) vs HM concentration (ng.g^−1^) of the first-year results and detecting inflation of the values when sample weight was <0.02 g (Appendix S1: Fig. S1). These findings and methods are consistent with previous studies indicating a ‘small sample effect’ in which fecal samples <0.02 g can produce anomalously inflated results (Millspaugh and Washburn, 2004; Hayward *et al.*, 2010; Ayres *et al.*, 2012).

We often collected repeated samples from the same individual per day. In order to prevent pseudoreplication, we only retained data from the sample with highest mass, since larger fecal samples are more likely to have good hormone detectability, repeatability and precision (Ayres *et al.*, 2012).

Metabolite extraction occurred by adding 90% methanol using binned solvent:sample ratios (Table 1). Previous studies on whale feces add the same solvent volume to all samples regardless of sample mass (e.g. Wasser *et al.*, 2000; Rolland *et al.*, 2005). However, given recent concerns about potential variation in extraction efficiency, we attempted to keep the solvent:sample ratio within a relatively narrow range. Samples were therefore divided into four mass bins, with solvent volume scaled down appropriately for the smaller mass. Solvent:sample ratios were maintained within a range of 1:10 to 1:25.

**Table 1:** Sample weighing and hormone extraction using binned solvent:sample ratios

Weight (g) range	Methanol added (μl)
0.02–0.04	500
0.05–0.09	1000
0.10–0.14	1500
0.15–0.20	2000

Tubes were loaded onto a plate shaker at 500 rpm for 30 min. Afterwards, they were centrifuged at 2200 rpm for 20 min (i.e. 285 RCF) and the supernatant (i.e. methanol containing the extracted material) was pipetted onto a microcentrifuge tube that was then dried down using pressurized air. Samples were then resuspended to their original volume in ultra-pure deionized water (1:1 dilution), with 10 min of sonication (ultrasonic cleaner, Cole-Palmer DTH) and 1 min vortexing to aid in resuspension. If particulates were still visible on the side of the tube, sonication and vortexing were repeated up to three times.

Seven fecal sample extracts were also pooled to conduct parallelism and accuracy tests (Table 2). Pooled samples were pipetted onto new microcentrifuge tubes, dried down and resuspended to their original volume, as described previously for the other samples. The pooled sample for the T3 assay was reconstituted with half the amount of deionized water, resulting in a 2-fold concentrated sample.

**Table 2:** Analytical validation pooled sample information and statistical results of assay validations with gray whale fecal extracts in progesterone, testosterone, cortisol and T3 immunoassays

Assay	Parallelism sample pool	Parallelism results	Accuracy sample pool	Accuracy results
Progesterone	*N* = 3 (A, B, C)	*F*_1,5_ = 1.253 *P* = 0.405	*N* = 3 (A, B, C)	Slope = 0.921 *r^2^* = 0.997
Testosterone	*N* = 7 (A, B, C, D, E, F, G)	*F*_1,4_ = 1.070 *P* = 0.431	*N* = 4 (B, E, F, G)	Slope = 0.966 *r*^2^ = 0.997
Cortisol	*N* = 5 (A, B, C, D, E)	*F*_1,6_ = 1.023 *P* = 0.500	*N* = 4 (A, B, C, F)	Slope = 1.269 *r*^2^ = 0.992
T3	*N* = 3 (E, F, G)	*F*_1,6_ = 1.386 *P* = 0.353	*N* = 3 (E, F, G)	Slope = 1.026 *r*^2^ = 0.996

*Legend: (A) Whale of undetermined maturity and sex with minimum age of 10 years in 2017 (whale ID: ErPNW-323), (B) resting female with minimum age of 21 years in 2017 (whale ID: ErPNW-19), (C) mature male with minimum age of 23 years in 2017 (whale ID: ErPNW-256), (D) mature whale of unknown sex with minimum age of 21 years in 2017 (whale ID: ErPNW-287), (E) immature male with minimum age of 1 year in 2016 (whale ID: ErPNW-223), (F) immature male of unknown age (ID: STRAND1), (G) immature male of unknown age (ID: STRAND2).*

**Figure 1:**
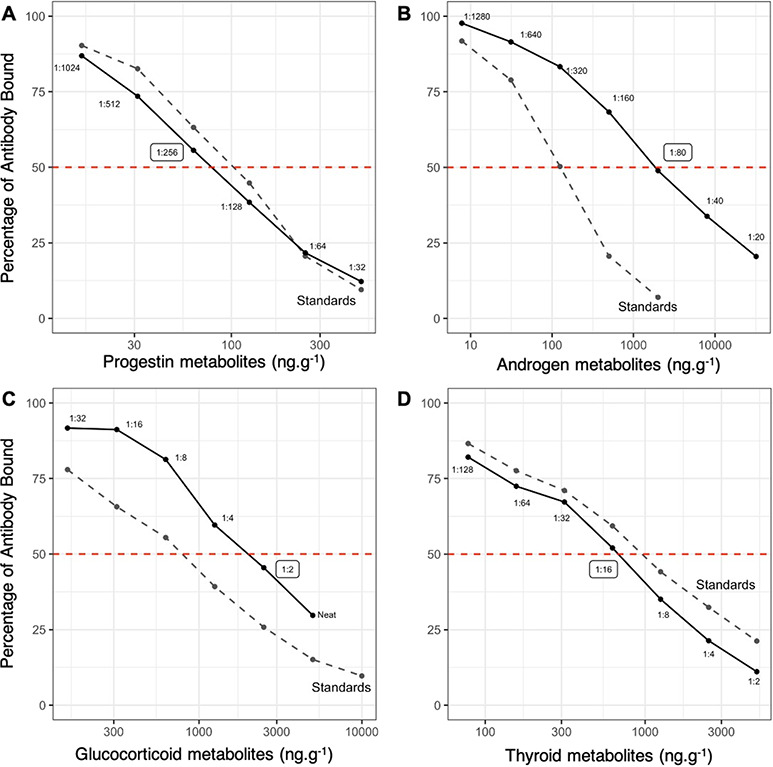
Parallelism plots for progestin, androgen, glucocorticoid and thyroid metabolite standard concentrations (gray dashed line) against serial diluted pooled gray whale fecal samples (black solid line). Accuracy dilutions were determined by 50% binding point (red dashed line) on the parallelism curve, indicated by circled dilution.

#### Hormone assays and analytical validations

Commercial enzyme-linked immunosorbent assay (ELISA) kits from Enzo Life Sciences were used to quantify immunoreactive progestin, androgen and GC metabolites (progesterone kit #ADI-900-011, testosterone kit #ADI-900-065 and cortisol kit #ADI-900-071; https://www.enzolifesciences.com), while thyroid metabolites were quantified using the T3 kit from Arbor Assays (T3 kit # K056-H1; https://www.arborassays.com).

Analytical validations included standard measures of parallelism and accuracy (Grotjan and Keel, 1996; Palme, 2019). Parallelism was analysed by running serial dilutions of the pooled samples in assay buffer provided in each kit. Six dilutions were used for the progesterone assay (1:32–1:1024), seven for the testosterone (1:20–1:1280) and seven for the T3 (1,2–1:128). The cortisol dilution set included a neat solution (non-diluted) and five dilutions ranging from 1:2–1:32. This test determined the dilution for a sample at 50% binding, which is the most accurate portion of the curve for measuring hormones. Accuracy was tested by spiking a set of hormone standards for each hormone assay with a pooled sample at a dilution of 50% binding, as indicated by the parallelism curve (Fig. 1). All pooled validation samples, standards and controls were assayed in duplicate for parallelism tests and in triplicate for accuracy tests. Inter-assay coefficient of variations (CVs) were required to be <15% and intra-assay CVs were required to be <10% for acceptance. All assay plates included a full standard curve, non-specific binding wells, total activity wells and blank wells.

Following validations, sample extracts were initially diluted to the 50% binding value determined from parallelism tests for each assay (Fig. 1). Thyroid metabolites were not quantified in 2016 due to the fact that thyroid assay validations consumed all available sample in this year. Samples were run in duplicate in 2016 and 2017 and in triplicate in 2018. All of the analyses followed the manufacturer’s protocol (Enzo, 2014, 2015a, 2015b; Arbor Assays, 2016). Values below the LOD were excluded from the analysis (Wood *et al.*, 2011).

The primary circulating GC in baleen whales is believed to be cortisol, but corticosterone is also detectable in some sample types (e.g. gray whale baleen; Hunt *et al.*, 2017a, 2017b). Furthermore, some mammalian fecal metabolites of cortisol bind well to corticosterone antibodies (Wasser *et al.*, 2000). Therefore, two ELISA GC kits (cortisol and corticosterone, from Enzo Life Sciences) were tested using five samples collected during a pilot field season in 2015 to determine which assay performs best for gray whale fecal samples. Our assumption was that the assay detecting greater concentrations of immunoreactive hormone metabolites was the superior assay (i.e. able to bind to a broader range of GC fecal metabolites). The cortisol assay produced highest apparent GC concentration, so all subsequent GC analyses utilized the cortisol assay.

### Physiological validations

Hormone metabolite concentration results were compared across the varied demographic units to conduct the physiological validations. HM concentrations were examined todetermine and differentiate between population baseline and individual HM concentrations that may indicate pregnancy, male reproductive activity, stress response or nutritional stress. Using all results for each HM, we calculated and plotted the probability density, mean, mean ± 1 standard deviation (SD) and the mean ± 2 SD.

Predictions of HM variation relative to physiological or reproductive state were based on findings from the literature, including higher progestin metabolite concentrations in pregnant females, higher androgen metabolite concentrations in mature males, higher GC metabolite concentrations in pregnant females compared to other demographic units (Rolland *et al.*, 2005), higher GC metabolite concentrations in known-stressed individuals (e.g. injured or entangled individual;Rolland *et al.*, 2005, 2017; Hunt *et al.*, 2006) and depressed thyroid metabolite concentrations with nutritional limitation (Behringer *et al.*, 2018).

For validation of our derived distributions and statistics for each HM, we compared the HM values of individual whales from ‘known specific states’ (sex, age class and reproductive status) to the population data through graphical overlays on the relevant plot: (1) known pregnant females (progestin metabolite; *N* = 4), (2) a case study of a mature male (whale ID: ErPNW-80) assumed to be in reproductively competitive behavior based on field observations of racing and breaching activity with another mature male as they followed a mature female (androgen metabolite; *N* = 1), (3) stranded whales (GC metabolite; *N* = 4) and a case study of an injured immature male (whale ID: ErPNW-223; *N* = 1) and (4) individuals of known-nutritional state based on drone photogrammetry results (Lemos et al., 2020; thyroid metabolite; good (*N* = 4) and poor body condition (*N* = 4)). Individuals from any demographic unit with fecal thyroid metabolite data from the same day as body condition assessment were selected to represent ‘good body condition’ whales if they had the four highest body area index (BAI) values, while the four lowest BAI values defined ‘poor body condition’ whales. BAI is a length-invariant index of whale body condition that can be directly compared between individuals of varying lengths and demographic units (Burnett *et al.*, 2019). While these hormone distribution and comparison cases are limited in number and cannot provide ‘absolute answers’ for physiological validations, they constitute all available comparison cases from the entire study period (including archived samples collected from stranded whales) and are valuable in this initial assessment of the potential of fecal hormone analytical methodology for gray whales.

### Statistical methods

Parallelism results were log-transformed and plotted as concentration versus percentage of antibody bound. Values were assessed with an *F*-test to determine the variance of slopes for each HM against the standard curve for the associated hormone. Accuracy results were plotted as known standard concentrations versus observed concentrations (i.e. pooled samples plus standards) and analysed by a linear regression, with acceptable accuracy limits of *R*^2^ > 0.95 and slope between 0.8 and 1.3 (Grotjan and Keel, 1996). These statistical tests were conducted using Microsoft Excel (Office 365, version 1808 1902) with an alpha set at 0.05.

A Shapiro–Wilk normality test was conducted to verify the distribution of the variables assessed in this study. Since all HM data displayed positively skewed distribution, we log-transformed (values +1) before conducting statistical analyses. This and the following statistical tests were conducted in R (version 3.5.0; R Core Team, 2019) with an alpha set at 0.05.

We conducted a series of linear mixed models (LMM) to assess the influence of multiple factors (demographic unit, day, month, year, study site and other HM) on HM concentrations, using the *lme4* package in R (Bates *et al.*, 2015). Some whales contributed more than one sample to the dataset on different days or in different years; due to potentially important variation in hormones across seasons and years, these samples were retained if they were collected on different days, but, in order to account for pseudoreplication, all models included whale identification as the random effect. Models were selected based on the Akaike’s information criterion (AIC; Burnham *et al.*, 2011). The LMM fit was estimated by calculating the marginal *R*^2^ (*R*^2^m; variance explained by fixed effects) and the conditional *R*^2^ (*R*^2^c; variance explained by fixed and random effects; entire model), using the *MuMIn* package (Nakagawa and Schielzeth, 2013; Barto, 2018). *F*-statistics and *P*-values were obtained using the *lmerTest* package (Kuznetsova *et al.*, 2017). A pairwise analysis of estimated marginal means (EMMs) was conducted to compare significant fixed effects, using the *emmeans* package in R (Lenth, 2019). Univariate supplementary tests were also conducted to verify linear correlations between HM in resting females and mature males over the three different sampling years, using the *lm* function in R.

## Results

A total of 171 whales were photo-identified throughout the study period, including 40 females, 40 males and 91 individuals of unknown sex. Sixteen of the sexed individuals were determined by fecal genetic analyses (see methods in Lemos *et al.*, 2020). Eleven mother–calf pairs were sighted during our study period, with the majority of the cases occurring in 2016 (*n* = 9), followed by one each in 2017 (*n* = 1) and 2018 (*n* = 1).

The review of photographs and drone recordings detected a moderate injury on an immature male (whale ID:ErPNW-223) on 20 June 2018, with apparent wounds from a propeller/vessel strike on its dorsal side (Appendix S1: Fig. S2). A fecal sample from this whale was collected in the same day at 12:26. Reviewed imagery of this individual on the previous day (19 June 2018 at 12:22) did not detect any sign of injury. Thus, it is assumed that whale ErPNW-223 endured this injury within 24 h of fecal sample collection on 20 June 2018. The review of drone recordings and behavior data also detected a mature male (whale ID: ErPNW-80) in reproductively competitive behavior with another male while following a known female, and a fecal sample from this individual was collected in the same day (28 August 2016). Therefore, we present these two individuals (ErPNW-223 and ErPNW-80) as case studies on typical endocrine profiles of physiologically stressed individuals and adult breeding males, respectively.

We collected a total of 158 fecal samples (2016: *n* = 53; 2017: *n* = 33; 2018: *n* = 72) from 69 different whales, totaling 40.12% of all individual whales sighted by our team. Twenty five of these samples were excluded from the analysis due to fecal sample mass below the acceptable threshold of 0.02 g. In addition, 17 samples were eliminated from the final dataset due to repeated samples per day per individual (*n* = 15) and to unknown individual origin (*n* = 2). Including the four stranded whale fecal samples (Appendix S1: Table S1), we conducted HM analyses on 120 fecal samples. Hormone metabolite concentrations below the LOD were excluded from statistical analyses. Thus, a final sample size of 114 for progestin metabolites, 101 for androgen metabolites, 92 for GC metabolites and 115 for thyroid metabolites were further assessed.

### Assay validations

All assays exhibited parallelism (Fig. 1) as the slope of serial dilutions was tested against the standard curves and were not statistically significant for either progesterone, testosterone, cortisol or T3 assays (Table 2). Accuracy slopes indicated no interference from the sample matrix in any of the four assays (Table 2). Inter- and intra-assay CVs are as follows, respectively: cortisol (8.42 and 10.30), testosterone (6.20 and 5.24), progesterone (10.10 and 5.92) and T3 (3.57 and 4.75).

### Fecal steroid and thyroid hormone metabolite variation

Fecal HM concentrations (ng.g^−1^, dried mass) varied across samples, with thyroid metabolites displaying the highest mean concentration (142.41 ± 320.28, range: 1.28–2134.86 ng.g^−1^), followed by progestin metabolites (P_m_; 71.46 ± 84.05, range: 0.02–488.05 ng.g^−1^), androgen metabolites (A_m_; 59.02 ± 189.32, range: 1.28–1478.04 ng.g^−1^), and finally GC metabolites (GC_m_; 16.51 ± 11.99, range: 3.24–70.22 ng.g^−1^; Table 3; Appendix S1: Fig. S3). HM concentrations in the water samples extracted from the desalting process were virtually undetectable, indicating no or very minimal sample HM loss.

**Figure 2:**
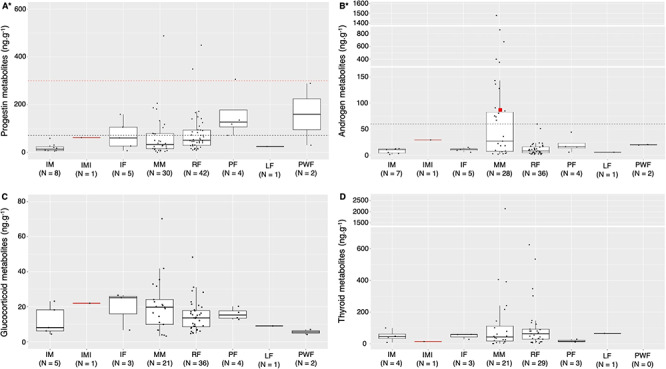
Variation in progestin (A), androgen (B), glucocorticoid (C) and thyroid (D) metabolite concentrations (ng.g^-1^, dried mass) by demographic units of gray whale fecal samples collected from May to October of 2016–2018 along the Oregon coast, USA. Each boxplot shows the interquartile range as the white box that encloses 25–75% of the data, the median as the horizontal black line, and the whiskers are the vertical black lines that extend from 5–95% of the data. Each black dot represents one data point used in the boxplot. Asterisks indicate the significance of demographic units on the respective hormone metabolite in the linear mixed models. X-axis groups: IM - immature male; IMI - immature male (injured whale, ID: ErPNW-223); IF - immature female; MM - mature male; RF - resting female; PF - pregnant female; LF - lactating female; PWF - postweaning female. The solid red line represents a case study of an injured immature male (whale ID: ErPNW-223) whose fecal sample was collected within 24 hours of a propeller/vessel strike injury and may reflect a typical endocrine profile of physiologically stressed individuals. (A) The dashed black line designates a putative threshold of 70.78 ng.g^-1^ for pregnancy in gray whales and the dashed red line designates a more conservative putative threshold of 300 ng.g^-1^ for pregnancy. (B) The white bars indicate breaks in the y-axis. The dashed black line designates a putative threshold of 60 ng.g^-1^ for maturity in male gray whales. The red square represents another case study of a mature male (whale ID: ErPNW-80) whose fecal sample was collected during a competitive behavior with another male and may reflect a typical endocrine profile of adult breeding males. (D) The white bar indicates a break in the y axis.

**Table 3:** Comparison of whale fecal hormone metabolites (ng.g^−1^, dried mass) by demographic units, including mean concentration ± standard deviation in the first row, median in the second row and range (min–max) in the third row. Rows in italics in the table include concentrations of stranded whales and case studies (endocrine profiles typical of physiologically stressed individuals and adult breeding males), and putative thresholds for pregnancies (progestin metabolites) and maturation in males (androgen metabolites)

Demographic unit	N	Progestin metabolites	N	Androgen metabolites	N	Glucocorticoid metabolites	N	Thyroid metabolites
Immature male	8	19.43 ± 18.05 13.11 (5.49–58.71)	7	8.14 ± 4.72 10.62 (1.44–12.54)	5	12.02 ± 8.21 8.11 (4.41–23.17)	4	49.58 ± 37.86 44.92 (8.44–100.02)
Mature male	30	69.77 ± 97.69 32.63 (0.016–488.05)	28	174.44 ± 328.11 70.32 (1.97–1478.04)	21	20.78 ± 15.72 19.76 (3.24–70.22)	21	192.57 ± 461.10 47.03 (1.28–2134.86)
Immature female	5	71.60 ± 61.65 60.34 (6.57–159.02)	5	10.63 ± 3.49 11.27 (5.65–15.01)	3	19.49 ± 11.10 25.19 (6.69–26.59)	3	48.42 ± 17.56 58.30 (28.14–58.82)
Resting female	42	78.37 ± 85.75 50.80 (8.71–449.07)	36	12.10 ± 12.54 8.18 (1.69–59.84)	36	14.80 ± 9.00 13.61 (10.48–48.30)	29	8 ± 152.97 63.55 (3.99–624.50)
Pregnant female	4	157.41 ± 102.62 126.49 (70.78–305.86)	4	20.47 ± 16.58 15.94 (5.71–44.27)	4	15.88 ± 3.38 15.27 (12.75–20.25)	3	18.57 ± 9.64 15.58 (10.77–29.35)
Lactating female	1	24.52	1	5.68	1	9.06	1	65.88
Postweaning female	2	159.36 ± 183.30 159.36 (29.75–288.97)	2	19.94 ± 0.89 19.94 (19.31–20.57)	2	5.61 ± 2.06 5.61 (4.16–7.07)	—	—
*Stranded whales*	*—*	*—*	*—*	*—*	*1* *1* *1* *1*	*23.60 (†)* 5.83 (*) *30.78 (¥)* *26.58 («)*	*—*	*—*
*Case studies*	*—* *—*	*—* *—*	*—* *1*	*—* *85.93 (€)*	*1* *—*	*22.01 (§)* *—*	*—* *—*	*—* *—*
*Putative thresholds*	*—*	*70.78 (¤)* *300.00 (‡)*	*—*	*60.00 (£)*	*—*	*—*	*—*	*—*

Legend: (†) Stranded immature male (STRAND 1); (*) Stranded immature male (STRAND 2); (¥) Stranded resting female (STRAND 3); («) Stranded immature female (STRAND 4); (§) injured immature male gray whale (whale ID: ErPNW-223); (€) mature male in competitive behavior (whale ID: ErPNW-80 T); (¤) putative threshold for pregnancy; (‡) conservative putative threshold for pregnancy; (£) putative threshold for maturation in male.

### Model selection

Sixteen different models were run to determine the influential predictor variables of progestin, androgen and GC metabolites, while only nine models were run for the thyroid metabolites (Appendix S1: Table S3). Since thyroid HMs were not quantified in 2016, the thyroid dataset was limited to only one study site (i.e. Newport). Thus, study site was not used as a fixed variable for thyroid metabolite models. Models were selected based on the lowest AIC, and *R*^2^ values from the selected models indicated that the variance in all HM models was better explained by both fixed and random effects (Appendix S1: Table S2).

In the progestin metabolite model (*R*^2^m = 0.70, *R*^2^c = 0.80), the predictor variables demographic unit (*F* = 4.98, *P* < 0.001, *df* = 9), year (*F* = 13.79, *P* < 0.001, *df* = 1) and androgen metabolites (*F* = 17.38, *P* < 0.001, *df* = 1) were significant, with androgen metabolites the most significant factor. The androgen metabolite model (*R*^2^m = 0.54, *R*^2^c = 0.87) included the significant variables demographic unit (*F* = 3.43, *P* < 0.01, *df* = 9) and progestin metabolites (*F* = 10.61, *P* < 0.01, *df* = 1). The significant predictor variables for the glucocorticoid metabolite model (*R*^2^m = 0.49, *R*^2^c = 0.64) were year (*F* = 17.44, *P* < 0.001, *df* = 1) and thyroid metabolites (*F* = 25.54, *P* < 0.001, *df* = 1). For the thyroid metabolite model (*R*^2^m = 0.66, *R*^2^c = 0.71) significant predictor variables were year (*F* = 26.25, *P* < 0.001, *df* = 1) and GC metabolites (*F* = 24.64, *P* < 0.001, *df* = 1). The GC metabolite model displayed the lowest *R*^2^ values compared to the other HM models, indicating that GC metabolites are likely more influenced by unmeasured external factors than the other HMs.

### Demographic unit comparisons

Based on EMM from LMM results, significant progestin metabolite differences were found between immature males and immature females (*P* < 0.05), and between pregnant females and immature males (*P* < 0.001), mature males (*P* < 0.01) and resting females (*P* < 0.05). We also found significant androgen metabolite differences between mature males and resting females (*P* < 0.05; Table 4).

The highest mean progestin metabolite concentration was observed in postweaning females, followed by pregnant females (Fig. 2A, Table 3). Mature males exhibited the highest mean concentration for androgen metabolites with a large interquartile range, followed by pregnant females (Fig. 2B, Table 3). The highest mean GC metabolite concentration was detected in mature males, followed by immature females (Fig. 2C, Table 3), while the highest mean for thyroid metabolite concentrations was observed in mature males, followed by a lactating female (Fig. 2D, Table 3). It is important to note variation in sample size for each demographic unit, with significantly larger sample sizes for mature males and resting females and therefore greater confidence in population estimates for these two demographic groups.

### Temporal variation of hormone metabolites

Although month did not have significant effects on any of the HM in the LMM (*P* > 0.05), year did have a significant effect on progestin (*P* < 0.01), GC (*P* < 0.001) and thyroid (*P* < 0.001) metabolite concentrations (Table 4).

### Hormone metabolite comparisons

**Figure 3:**
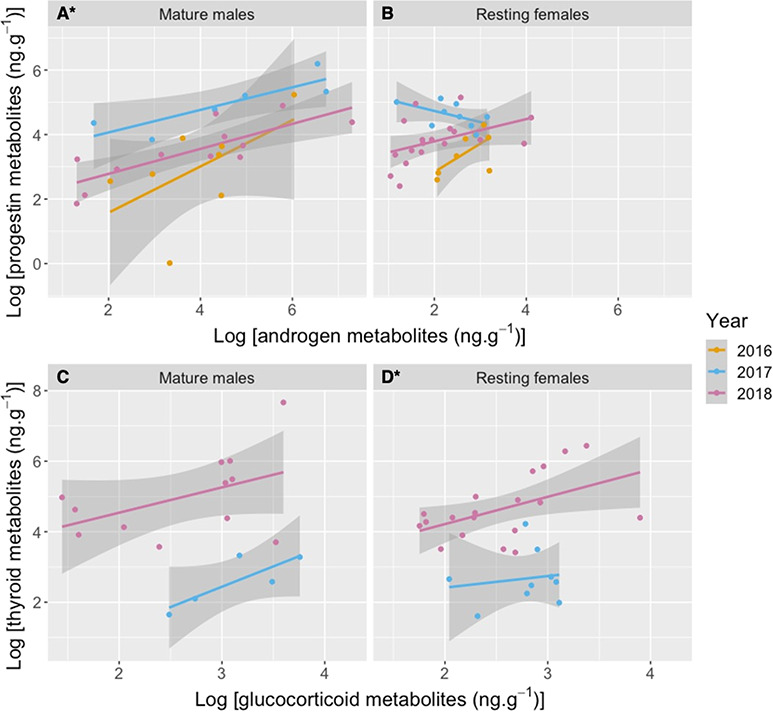
Linear correlations by year between hormone metabolite concentrations (ng.g^-1^, dried mass) of gray whale fecal samples collected from June to October of 2016–2018 along the Oregon coast, USA: androgen vs progestin metabolites in mature males (A) and resting females (B), and glucocorticoid vs thyroid metabolites in mature males (C) and resting females (D). Asterisks indicate significant correlations between hormone metabolites in specific years. Fecal thyroid metabolite analysis was not performed in 2016. Note logarithmic scale of x- and y-axes.

Significant correlations were found between progestin and androgen (LMM: *P* < 0.001; Fig. 3A), and between GC and thyroid metabolite concentrations (LMM: *P* < 0.001; Fig. 3B). Linear regressions between HMs in both resting females and mature males indicated a significant correlation between progestin and androgen metabolites in mature males in the years of 2017 (rate of change = 0.35, *F*_1,4_ = 10.39, *P* < 0.05, *R*^2^ = 0.65) and 2018 (rate of change = 0.39, *F*_1,10_ = 18.51, *P* < 0.01, *R*^2^ = 0.61). A significant correlation was also determined between GC and thyroid metabolites in resting females in 2018 (rate of change = 0.32, *F*_1,17_ = 5.71, *P* < 0.05, *R*^2^ = 0.21).

### Physiological validations

**Figure 4:**
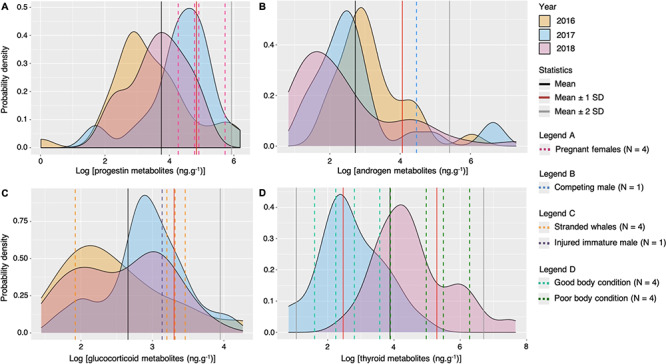
Probability density plots of gray whale log fecal hormone metabolite (ng.g^-1^, dried mass) variability by years collected from May to October of 2016–2018 along the Oregon coast, USA. Fecal thyroid metabolite analysis was not performed in 2016. Statistics (mean, mean ± 1 standard deviation, and mean ± 2 standard deviations of all values) are indicated in solid lines. The statistics mean – 1 standard deviation and mean – 2 standard deviations were only performed in the thyroid metabolite analysis. Dashed lines indicate individuals of ‘known state’ and vary according to the hormone metabolites: (A) log progestin metabolite levels with documented pregnant females over the study period identified, (B) log androgen metabolite levels with a mature male in a competing behavior (whale ErPNW-80) identified, (C) log glucocorticoid metabolite levels with four stranded gray whales (Table 1) and an immature male injured within 24 hours of fecal sample collection (whale ErPNW-223, Appendix C - S1, Fig. 3.S3) identified, and (D) log thyroid metabolite levels with whales of documented good and poor body condition (based on Lemos et al. 2020) identified. Note logarithmic scale of x-axes.

Progestin metabolite concentrations of pregnant females were significantly different from resting females, mature males and immature males (Table 4). In addition, all four pregnant females analysed in this study exhibited values above the mean progestin metabolite concentrations in mature individuals, with two of the values above the first SD (Fig. 4A). Based on these results, we propose two putative progestin thresholds to determine pregnancy in gray whales. The first tentative threshold is of 70.78 ng.g^−1^ (i.e. 4.27 on the log +1 scale) that encompasses all identified pregnant females in this study. The second threshold is of 300 ng.g^−1^ (i.e. 5.71 on the log +1 scale), which is a more conservative limit that maximizes specificity (i.e. minimizes false-positive pregnancies). The first putative threshold lies above the mean, while the second threshold lies above the first SD above the mean (Fig. 2A and Fig. 4A). While progestin metabolite values of three of the four pregnant females fall below this conservative threshold, the fourth pregnant female exhibited a relatively high progestin metabolite level (whale ID:ErPNW-14; 305.856 ng.g^−1^; log (305.856 + 1) = 5.73). Whales with greater progestin values than whale ErPNW-14 came from one mature male with simultaneously high androgen values (695.37 ng.g^−1^) and two resting females that may constitute misidentified pregnancies.

Androgen metabolite concentrations of mature males were significantly different from those of resting females, but not from any other demographic unit (Table 4). Despite the large range in androgen metabolites in mature males (1.97–1478.04 ng.g^−1^; Table 2), no individual from another demographic unit exhibited values greater than 60 ng.g^−1^, thus indicating a potential threshold value for determining maturity in gray whale males (Fig. 2B). A fecal sample from a mature male assumed to be reproductively competing with another male based on behavior data (whale ID:ErPNW-80; sampled on 28 Aug 2016) exhibited a relatively high androgen metabolite concentration (85.932 ng.g^−1^; log (85.932 + 1) = 4.46), falling between the first and second SD (Fig. 4B). Using this androgen value from the whale ErPNW-80 as a case study that may reflect a typical endocrine profile of adult breeding males, our results indicate that nine fecal samples (10.71% of 84 samples) from seven different individuals fall above this value, all of which were mature males.

**Table 4:** Significant pairwise comparisons of estimated marginal means (EMMs) of the selected linear mixed models for gray whale hormone metabolite concentrations

Hormone metabolites	Significant predictors of the selected models	Estimate	Standard error	Degrees of freedom	T ratio	*P* value
Progestin metabolites	***Demographic unit*** Immature male—immature female Immature male—pregnant female Mature male—pregnant female Resting female—pregnant female ***Year*** 2017–2018	−1.8752 −2.3562 −1.7479 −1.2985 1.07	0.485 0.479 0.387 0.359 0.305	34.1 32.6 27.6 25.2 37.4	−3.863 −4.915 −4.513 −3.621 3.520	0.0150 0.0009 0.0036 0.0349 0.0012
Androgen metabolites	***Demographic unit*** Mature male—resting female	1.7728	0.458	27.9	3.868	0.0179
Glucocorticoid metabolites	***Year*** 2017–2018	1.04	0.26	33.9	3.990	0.0003
Thyroid metabolites	***Year*** 2017–2018	−2.39	0.493	36.4	−4.849	<0.0001

Glucocorticoid metabolite concentrations did not significantly vary by demographic unit. Our case study of an identified injured whale (whale ID: ErPNW-223) indicates a GC concentration that may reflect a typical endocrine profile of a physiologically stressed gray whale. Three fecal samples were collected from this whale over the course of our study with the following GC metabolite concentrations: 8.11 ng.g^−1^ on 28 August 2016 (uninjured), 4.41 ng.g^−1^ on 1 July 2017 (uninjured) and 22.01 ng.g^−1^ on 20 June 2018 (injury present and known to have occurred within the previous 24 h). This injury-related GC level is almost three times higher than the values previously reported for the same individual during assumed non-stress-related sampling periods and also falls just below the first SD of GC values for the population of sampled whales (Fig. 4C). Three of the four fecal samples from stranded gray whales, which are assumed to have concentrations that may reflect typical endocrine profiles of physiologically stressed individuals, had GC metabolite values near or above the first SD.

When comparing the GC metabolite concentration from the injured whale (ErPNW-223; 22.011 ng.g^−1^; log (22.011 + 1) = 3.14) to other whales (Fig. 2C), only 15 values in our dataset (17% of 88 samples) fall above this value, including the injured individual. Interestingly, of these whales with values above this GC concentration, three were mature males and one resting female that also displayed high androgen or progestin metabolite values respectively, all of which also fell above the putative androgen or progestin metabolite thresholds.

Thyroid metabolite concentrations were also not significantly different between demographic units. Yet, whales with good body condition (as determined by Lemos *et al.*, 2020) consistently exhibited lower thyroid metabolite concentrations compared to whales with poor body condition, and all four T3 values from the whales in good body condition fell below the population mean (Fig. 4D). Moreover, three of four whales with poor body condition displayed relatively high thyroid metabolite concentrations and fell above the population mean. Individuals with a good body condition displayed thyroid metabolite concentrations that ranged from 3.989 to 34.560 ng.g^−1^, while individuals with a poor body condition exhibited a range of 48.103 to 534.281 ng.g^−1^.

## Discussion

This study represents the first endocrine assessment in ENP gray whales and advances our understanding of baleen whale hormone variation relative to physiological state. This assessment is unique and offers useful findings because the study comprised a relatively large sample size of fecal HM concentrations from the sample population (28–34% of the PCFG based on the current abundance estimate; Calambokidis and Perez, 2017), which are paired with individual data on whale demographic unit, reproductive state, behavioral information and health status. We acknowledge our low sample size for some demographic units and for our physiological validations of pregnant, mature, stressed and reproductively competing males. Differentiating baseline from abnormal values in whale HM concentrations based on physiological validations is challenging due to the opportunistic and rare nature of sampled events, making them even more critical to describe when captured. Our putative thresholds and case studies provide a starting place toward a better understanding of baseline, variation and deviant HM concentrations in baleen whales and will contribute to future endocrine assessments. Through continued sampling and monitoring of a well-studied population such as these PCFG gray whales, more samples can be obtained and analysed to further inform and clarify baleen whale physiological HM patterns.

### Reproductive hormone metabolites

Previous studies have reported higher fecal progestin metabolite concentrations in pregnant females, followed by lactating females (Valenzuela-Molina *et al.*, 2018; Hunt *et al.*, 2019) or mature males (Rolland *et al.*, 2005). In this study, we also found higher mean values in pregnant and postweaning females; however, the highest three progestin values were observed in a mature male and in two resting females. Interestingly, this mature male also exhibited one of the highest androgen metabolite concentrations of this assessment. Progesterone is rarely assayed in male mammals, under the assumption that it is not informative in males, but scattered data indicate that it may not be uncommon for males to have high progesterone (Rolland *et al.*, 2005; K. Hunt, *pers. obs.*) and that progesterone may even play a direct role in male behavior in some mammals (Wagner, 2006; Bychowski and Auger, 2012). Progesterone is also a precursor for synthesis of all other steroid hormones, which may explain the high values observed for both reproductive HM concentrations in this individual (Goodman, 2009). Although the two resting females with high progestin concentrations were seen in the following season unaccompanied by a calf, these sightings occurred later in the season (September) so weaning events may have already occurred. Additionally, these females could have experienced miscarriages, pseudopregnancies and extended luteal phases or lost their calves for some other reason (Bergfelt *et al.*, 2011; Pallin *et al.*, 2018; Robeck and O’Brien, 2018; Hunt *et al.*, 2019). These undetected pregnancies are known phenomena that can generate inherent logistical problems with development of pregnancy diagnostics in whale studies (Hunt *et al.*, 2019).

Although all pregnant whales displayed progestin metabolite values above the population mean, individuals from other demographic units also exhibited similar concentrations. Thus, we have determined two putative pregnancy thresholds: the first threshold of 70.78 ng.g^−1^ encompasses all pregnant females, and a second threshold of 300 ng.g^−1^ that is more conservative and based on the highest progestin metabolite value for a known pregnant whale, which was clearly higher than the majority of other demographic units. This conservative threshold value is 3-fold higher than the mean for other mature females, which is consistent with the findings of other studies that described higher progestin metabolite concentrations in pregnant baleen whales compared to other mature females (Mansour *et al.*, 2002; Rolland *et al.*, 2005; Pallin *et al.*, 2018; Valenzuela-Molina *et al.*, 2018; Hunt *et al.*, 2019). Fecal progestin metabolite concentrations varied several orders of magnitude across the different mysticete species, which may be correlated to species-specific hormone secretion rates, hormone clearance rates, diet, digestive physiology, gut microbiota and mating strategies, as well as differences in assay antibody binding affinity for specific fecal metabolites. Other possible explanations include stage of gestation when fecal sample collection occurred. We believe our samples were collected in mid-gestation and progesterone has been shown in some species to steadily increase throughout gestation (Goodman, 2009). Thus, it is possible that progestin metabolite concentrations are still low during the first 6–8 months of pregnancy, as gestation in gray whale females last ~13 months (Rice and Wolman, 1971).

Fecal androgens were highest in mature males, followed by pregnant females, with the former displaying an 8-fold higher mean than the latter. These patterns match our predictions and are similar to patterns seen in NARWs (Rolland *et al.*, 2005). When comparing to immature males, mature males exhibited a 21-fold higher mean, likely reflecting enhanced gonadal steroidogenesis that characterizes sexual maturation (Rolland *et al.*, 2005). No demographic unit other than mature males displayed androgen metabolite concentrations higher than 60 ng.g^−1^. Thus, we were able to use this value as a putative threshold for determining maturity in gray whale males. In addition, as we observed a mature male in a reproductively competitive behavior (whale ID: ErPNW-80), and we postulate that its androgen metabolite concentration is typical of adult breeding males. Even though this is a case study of a single individual, its androgen concentration concurs with our putative male maturity threshold (60 ng.g^−1^), offering a physiological validation.

Significant correlations between progestin and androgen metabolite concentrations were documented in mature males in 2017 and 2018, and 2016 data illustrate the same trend (Fig. 4A). However, resting females displayed variable trend between progestin and androgen over the sampling years and this result may be associated with their reproductive cycle. Gray whales are known to have a 2-year reproductive cycle and to normally come into estrus every other year (Rice and Wolman, 1971). Thus, progestin metabolites may peak in alternate years for each female. Therefore, the alternating yearly trends in progestin and androgen metabolite concentrations may reflect a sampling bias toward one cohort of reproductive females, or a population-level pattern in reproductive activity driven by intrinsic cycles or environmental conditions (Lemos *et al.*, 2020). In order to verify this hypothesis, we recommend further monitoring of this gray whale population to investigate pregnancies and/or inter-calving intervals along the Oregon coast.

### Stress and nutritional stress-related hormone metabolites

No significant differences in gray whale GC metabolite concentrations were detected between demographic units, which is in line with the results of an assessment of blue whale fecal HM (Valenzuela-Molina *et al.*, 2018). However, that study performed both cortisol and corticosterone assays. While they observed no significant differences when using the cortisol antibody, differences among demographic units were detected with the corticosterone antibody. Through high-performance liquid chromatography (HPLC) analysis, Valenzuela-Molina *et al.* (2018) identified corticosterone (or a closely similar co-eluting metabolite) as the primary fecal GC metabolite in blue whales; 68% of the putative ‘cortisol’ detected by their cortisol assay co-eluted with a pure corticosterone tracer during HPLC, rather than co-eluting with a pure cortisol tracer. In other words, the assay antibody, though originally raised against a cortisol antigen, also binds to other GC fecal metabolites. Some species may have an even more complex array of fecal metabolites; Hunt *et al.* (2006) found that a different GC antibody (originally raised against a corticosterone antigen) detected at least nine different fecal metabolites in NARW feces, none of which co-eluted with pure cortisol or pure corticosterone. Studies in terrestrial species confirm that mammals commonly produce a wide assortment of fecal metabolites of a single parent hormone, and, further, that each assay antibody has a unique pattern of cross-reactivity to a given set of fecal metabolites (Palme, 2019). It is for this reason that fecal hormone analyses focus on relative patterns (e.g. comparison to case studies) rather than absolute values. We recommend that future assessments of GC metabolite concentrations in gray whales using immunoassay methodology should consider performing a HPLC or mass spectrometry analysis to further identify the composition of gray whale fecal hormone metabolites. Researchers should also consider using both corticosterone and cortisol kits, as well as specialty antibodies raised against common fecal GC metabolites of artiodactyls (e.g. 11-oxo-etiocholanolone; Palme, 2019) to identify the primary fecal GC metabolite in gray whales and provide increased clarity in variation between demographic units.

Our findings indicated higher mean GC metabolite concentrations in mature males, consistent with patterns seen in both NARW (Hunt *et al.*, 2006) and humpback whales (Hunt *et al.*, 2019). The high GC metabolite concentrations in mature males found in this study may be associated with intense reproductive competition (Hunt *et al.*, 2006), as commonly documented in several other mammals (Sands and Creel, 2004; Higham *et al.*, 2013).

The identified injured whale (ID: ErPNW-223) had fecal GCs above 76% of all GC values from the sampled gray whale population (Fig. 4B) and exhibited a within-individual elevation corresponding to the time of injury (i.e. as compared to samples from the same individual on prior days). The exact time of the propeller/vessel strike is unknown but based on the sighting time from the previous day where no injuries were observed, it occurred within 24 h of the fecal sample collection. Thus, it is possible that the lag time between the GC hormone release caused by the injury to the appearance (or peak) of the hormone signature in feces in gray whales is <24 h, a temporal scale correspondent to previous reports for mammals (Palme *et al.*, 1996, 2005; Wasser *et al.*, 2010).

Fecal samples from the four stranded whales add to our validation efforts of GC metabolite concentrations. Three stranded whale GC values fell well above the population mean and above the GC value from the whale ErPNW-223 when injured, and two were above the first SD. Intriguingly, *STRAND 2* (i.e. an entangled immature male; Appendix S1: Table 1) exhibited a very low GC metabolite concentration when compared to the other stranded whales (at least 4-fold lower). A low GC metabolite concentration in association with a stressful condition like an entanglement may indicate an impaired response by the hypothalamic–pituitary–adrenal axis due to chronic stress or adrenal fatigue (Norris, 2000; Cyr and Romano, 2009; Hunt *et al.*, 2013; Ajó *et al.*, 2018).

In addition to GC, thyroid hormones can also indicate nutrition-related stress (Jeanniard du Dot *et al.*, 2009; Ayres *et al.*, 2012; Spitz *et al.*, 2015). Several studies have reported low thyroid hormone concentrations in nutritionally stressed individual marine mammals, including Steller sea lions (*Eumetopias jubatus*; Jeanniard du Dot *et al.*, 2009) and killer whales (Ayres *et al.*, 2012). Yet, studies on fecal thyroid hormone metabolites in baleen whales are scarce, so far limited to a single assessment on humpback whales (Hunt *et al.*, 2019). Therefore, there are significant data gaps about how fecal thyroid HMs behave in baleen whales. Findings from Ayres *et al.* (2012) indicate a positive association between prey availability and thyroid metabolite concentrations in killer whales, but their data are also consistent with a seasonal effect and/or a thermoregulatory effect. In contrast, our gray whale assessment did not document a similar positive pattern between nutritional state and thyroid metabolite concentrations, and also showed no relationship with month. Using body condition data quantified by drone-based aerial photogrammetry (Lemos *et al.*, 2020) of fecal sampled gray whales, we show that whales with good body condition consistently exhibited lower thyroid hormone values, with all values below the population mean, and whales with a poor body condition displayed higher thyroid hormone concentrations, with all values above the population mean.

Our contrasting results may be due to the fact that thyroid hormones are also known to be closely related to metabolic activities, including thermoregulation and carbohydrate utilization (Behringer *et al.*, 2018). Previous studies on mammals have described negative correlations between thyroid hormones and ambient temperature, and also between thyroid hormones and body condition. For example, Oki and Atkinson (2004) reported high plasma thyroid hormones in adult harbor seals (*Phoca vitulina*) during the winter season. Another study described high plasma thyroid hormones in free-living arctic ground squirrels (*Urocitellus parryii*) during lactation, an energetically costly life stage, and low plasma thyroid hormones prior to hibernation when metabolic rate is depressed (Wilsterman *et al.*, 2015). Thus, thermoregulation could explain the high thyroid metabolite concentrations in gray whales with poor body condition. Blubber acts as an insulator in cetaceans, thus individuals with poor fat stores could need to maintain a higher metabolic rate, via secretion of greater circulating thyroid hormones in order to produce sufficient heat and maintain normal body temperature (Parry, 1949). However, it is debatable if baleen whales have difficulty maintaining their thermal homeostasis since whales have a very low thermal conductance due to their large size, mass and surface area (Lavigne *et al.*, 1990). In addition, in this study, sampling occurred during summer, when ocean temperatures were approaching their seasonal maximum. Therefore, it is unlikely that thermoregulation is the explanatory factor for the patterns seen herein for thyroid metabolite concentrations. We recommend measurements of the lower thermal conductance in gray whales to better understand this relationship between body condition and thyroid metabolite concentrations. Another potential explanation for the observed relationship between thyroid HM concentrations and whale body condition could be carbohydrate utilization, as thyroid hormones are known to increase with poor carbohydrate content in the diet, and poor nutritional state (Goodman, 2009).

Even though positive trends between GC and thyroid metabolite concentrations were observed in both years in resting females and mature males, supplementary linear regressions indicated a significant correlation between the HM concentrations in resting females in 2018 only. As described by Lemos et al. (2020), this population of PCFG gray whales exhibited poor body condition in 2017 that correlated to carry-over effects of poor foraging conditions the previous year. Thus, nutritional stress may have impacted gray whale HM concentrations in 2017 and the overall linear correlation results. Therefore, we recommend continued monitoring of this PCFG gray whale population to verify and further describe correlations between body condition and fecal HM physiology.

We also acknowledge a methodological limitation in our assessment of thyroid HM variation in gray whales that should be addressed in future studies. Fecal thyroid metabolites are now known to not attain 100% extraction efficiency in most alcohol extraction methods, as it will typically saturate the extraction solvent (Wasser *et al.*, 2010; Ayres *et al.*, 2012). Even though we devised different approaches to minimize the issue (e.g. multiple rounds of high-volume extraction in an effort to extract 100% of thyroid metabolites and reduction of variation in solvent:sample ratio so as to attain similar, though not 100%, extraction efficiency across samples), it has not been totally eliminated. Thus, it is best to focus on relative patterns in thyroid concentrations and not absolute values.

## Conclusions

In this study we successfully used fecal samples from gray whales as a non-invasive monitoring tool to advance our knowledge of baleen whale physiology, including documentation of HM variation relative to demographic units, years and other HM. As is typical in whale physiological research, our sample sizes of known-physiological-state individuals are often too low to allow confident conclusions, yet our contextualized dataset enabled us to propose tentative HM thresholds in gray whales for the identification of pregnancies and maturity in males. In addition, although the two case studies reported here (injured whale ErPNW-223, and mature male in reproductive competition ErPNW-80) cannot be used to definitively define absolute thresholds of fecal HM concentrations due to very low sample size, these individual case studies are valuable reference points. Furthermore, these case studies may prove informative for future research on endocrine profiles typical of adult breeding males and of physiologically stressed individuals.

Differentiating between baseline and abnormal concentrations is an essential first step to determining associations between deviant values and environmental pressures that may act as both acute and chronic disruptive factors. We encourage continued observation and sampling of this population of gray whales, and other baleen whales, to refine these threshold estimates, as well as HM correlations to each other and demographic units. The development of this knowledge will enable potential discrimination of disturbance events, mortality causes and impacts on population dynamics, thus informing decision-making processes regarding the conservation and management of threatened and endangered whales.

## Funding

This work was supported by the NOAA National Marine Fisheries Service Office of Science and Technology Ocean Acoustics Program [2017; 50-27], Oregon Sea Grant Program Development funds [2018; RECO-40-PD] and the Oregon State University Marine Mammal Institute. We are thankful for the support of Brazil’s Science Without Borders program, Brazil’s CNPq and the Harvard Laspau Institute for financial aid and academic advising to LSL. We are also thankful for the support of the Mamie Markham Research Award (Hatfield Marine Science Center/OSU) and Cetacean Society International for awards given to LSL to support data and laboratory analyses.

## Supplementary Material

Supporting_Information_coaa110
