# Supplementary material for: Assessment of fecal steroid and thyroid hormone metabolites in eastern North Pacific gray whales
Source: Conserv Physiol. 2020 Dec 7;8(1):coaa110. doi: 10.1093/conphys/coaa110 (PMC7720082; doi:10.1093/conphys/coaa110)
Supplement: Supporting_Information_coaa110 [file supporting_information_coaa110.docx]

**Conservation Physiology**

**Assessment of fecal steroid and thyroid hormone metabolites in eastern north Pacific gray whales**

Leila S. Lemos ^1*^, Amy Olsen ^2^, Angela Smith ^2^, Todd E. Chandler ^1^, Shawn Larson ^2^, Kathleen Hunt ^3^, Leigh G. Torres ^1^

^1^ Marine Mammal Institute, Fisheries and Wildlife Department, Oregon State University, 2030 SE Marine Science Dr, Newport, OR, 97365, USA

^2^ Conservation Programs and Partnerships, Seattle Aquarium, 1483 Alaskan Way, Seattle, WA, 98101, USA

^3^ Smithsonian-Mason School of Conservation, 1500 Remount Road, Front Royal VA 22630, USA

**Supporting Information**

**Appendix S1**

**Tables**

Table S1: General information of the four stranded gray whales along the Oregon and Washington coasts, USA, with fecal samples analyzed in this study.

| **Sample Id** | **Date of Collection** | **Location** | **Sex** | **Age class** | **Length (cm)** | **Condition** | **Marks** |
| --- | --- | --- | --- | --- | --- | --- | --- |
| **STRAND 1**  **STRAND 2**  **STRAND 3**  **STRAND 4** | 23-Mar-2016  15-Apr-2018  04-Jun-2018  06-Feb-2019 | Lincoln City, OR  Leadbetter Point State Park, WA  Florence, OR  Bandon, OR | Male  Male  Female  Female | Yearling  Yearling  Adult  Yearling | 973  804  1184  960 | Fresh dead  Advanced decomposition (stage 4)  Moderate decomposition  Advanced decomposition (stage 4) | -  Entanglement marks  Signs of killer whale attack  - |

Table S2: Generalized linear mixed model selection parameters of gray whale fecal hormone metabolites relative to the predictor variables demographic unit (DU), day of the year (DOY), month, year, study site and other hormone metabolites (progestin [P], androgen [A], glucocorticoids [GC], and thyroid [T] metabolites). All models used whale identification (ID) as a random effect. Models in bold were selected as the best models based on the Akaike Information Criterion (AIC). Marginal R^2^ (R^2^m) indicates the variance explained by fixed effects, and the conditional R^2^ (R^2^c) indicates the variance explained by both fixed and random effects (i.e., entire model). Since thyroid hormone metabolites were not quantified in 2016, the dataset was limited to only one study site (i.e., Newport). Thus, study site could not be used as a fixed variable for thyroid metabolite models.

| **Models** | **Degrees of freedom** | **AIC** | **R^2^m** | **R^2^c** |
| --- | --- | --- | --- | --- |
| ***Progestin metabolites (log):***  (1) logP ~ DU + DOY + month + year + study site + (1\|ID)  (2) logP ~ DU + DOY + month + year + (1\|ID)  (3) logP ~ DU + month + year + (1\|ID)  (4) logP ~ month + year + (1\|ID)  (5) logP ~ DOY + month + year + (1\|ID)  (6) logP ~ DOY + month + year + study site + (1\|ID)  **(7) logP ~ DU + DOY + month + year + logA + logGC + logT + (1\|ID)**  (8) logP ~ DU + DOY + month + year + logA + logGC + study site + (1\|ID)  (9) logP ~ DU + DOY + month + year + logA + logGC + (1\|ID)  (10) logP ~ DU + DOY + month + year + logA + study site + (1\|ID)  (11) logP ~ DU + DOY + month + year + logA + (1\|ID)  (12) logP ~ DU + DOY + month + year + logGC + study site + (1\|ID)  (13) logP ~ DU + DOY + month + year + logGC + (1\|ID)  (14) logP ~ DU + DOY + month + year + logT + (1\|ID)  (15) logP ~ DU + DOY + month + year + logA + logT + (1\|ID)  (16) logP ~ DU + DOY + month + year + logGC + logT + (1\|ID) | 22  21  20  10  11  12  **22**  24  23  23  22  23  22  20  21  21 | 337.5372  337.9848  329.7763  332.0831  340.8166  337.3327  **131.3517**  170.8632  171.4074  248.2457  246.3720  224.2423  223.0992  205.4724  161.9826  163.3408 | **0.70** | **0.80** |
| ***Androgen metabolites (log):***  (1) logA ~ DU + DOY + month + year + study site + (1\|ID)  (2) logA ~ DU + DOY + month + year + (1\|ID)  (3) logA ~ DU + month + year + (1\|ID)  (4) logA ~ month + year + (1\|ID)  (5) logA ~ DOY + month + year + (1\|ID)  (6) logA ~ DOY + month + year + study site + (1\|ID)  **(7) logA ~ DU + DOY + month + year + logP + logGC + logT + (1\|ID)**  (8) logA ~ DU + DOY + month + year + logP + logGC + study site + (1\|ID)  (9) logA ~ DU + DOY + month + year + logP + logGC + (1\|ID)  (10) logA ~ DU + DOY + month + year + logP + study site + (1\|ID)  (11) logA ~ DU + DOY + month + year + logP + (1\|ID)  (12) logA ~ DU + DOY + month + year + logGC + study site + (1\|ID)  (13) logA ~ DU + DOY + month + year + logGC + (1\|ID)  (14) logA ~ DU + DOY + month + year + logT + (1\|ID)  (15) logA ~ DU + DOY + month + year + logP + logT + (1\|ID)  (16) logA ~ DU + DOY + month + year + logGC + logT + (1\|ID) | 22  21  20  10  11  12  **22**  24  23  23  22  23  22  20  21  21 | 314.6159  313.8047  305.3878  325.1746  333.5905  334.7138  **179.4404**  215.0093  216.2330  279.4991  278.0867  238.6152  238.9865  217.6168  197.7768  196.2932 | **0.54** | **0.87** |
| ***Glucocorticoid metabolites (log):***  (1) logGC ~ DU + DOY + month + year + study site + (1\|ID)  (2) logGC ~ DU + DOY + month + year + (1\|ID)  (3) logGC ~ DU + month + year + (1\|ID)  (4) logGC ~ month + year + (1\|ID)  (5) logGC ~ DOY + month + year + (1\|ID)  (6) logGC ~ DOY + month + year + study site + (1\|ID)  **(7) logGC ~ DU + DOY + month + year + logP + logA + logT + (1\|ID)**  (8) logGC ~ DU + DOY + month + year + logP + logA + study site + (1\|ID)  (9) logGC ~ DU + DOY + month + year + logP + logA + (1\|ID)  (10) logGC ~ DU + DOY + month + year + logP + study site + (1\|ID)  (11) logGC ~ DU + DOY + month + year + logP + (1\|ID)  (12) logGC ~ DU + DOY + month + year + logA + study site + (1\|ID)  (13) logGC ~ DU + DOY + month + year + logA + (1\|ID)  (14) logGC ~ DU + DOY + month + year + logT + (1\|ID)  (15) logGC ~ DU + DOY + month + year + logP + logT + (1\|ID)  (16) logGC ~ DU + DOY + month + year + logA + logT + (1\|ID) | 22  21  20  10  11  12  **22**  24  23  23  22  23  22  20  21  21 | 216.3550  214.9332  205.5074  192.3020  201.9216  203.8289  **123.0816**  171.4304  170.2195  192.2396  190.4762  180.9093  179.5734  166.0224  145.0182  137.5646 | **0.49** | **0.64** |
| ***Thyroid metabolites (log):***  (1) logT ~ DU + DOY + month + year + (1\|ID)  (2) logT ~ DU + month + year + (1\|ID)  (3) logT ~ month + year + (1\|ID)  (4) logT ~ DOY + month + year + (1\|ID)  **(5) logT ~ DU + DOY + month + year + logP + logA + logGC + (1\|ID)**  (6) logT ~ DU + DOY + month + year + logP + logA + (1\|ID)  (7) logT ~ DU + DOY + month + year + logP + (1\|ID)  (8) logT ~ DU + DOY + month + year + logP + logGC + (1\|ID)  (9) logT ~ DU + DOY + month + year + logA + logGC + (1\|ID) | 19  18  9  10  **22**  21  20  21  21 | 241.5680  238.9924  241.7209  245.6578  **177.1910**  213.2809  230.1621  197.8141  184.6215 | **0.66** | **0.71** |

**Figures**


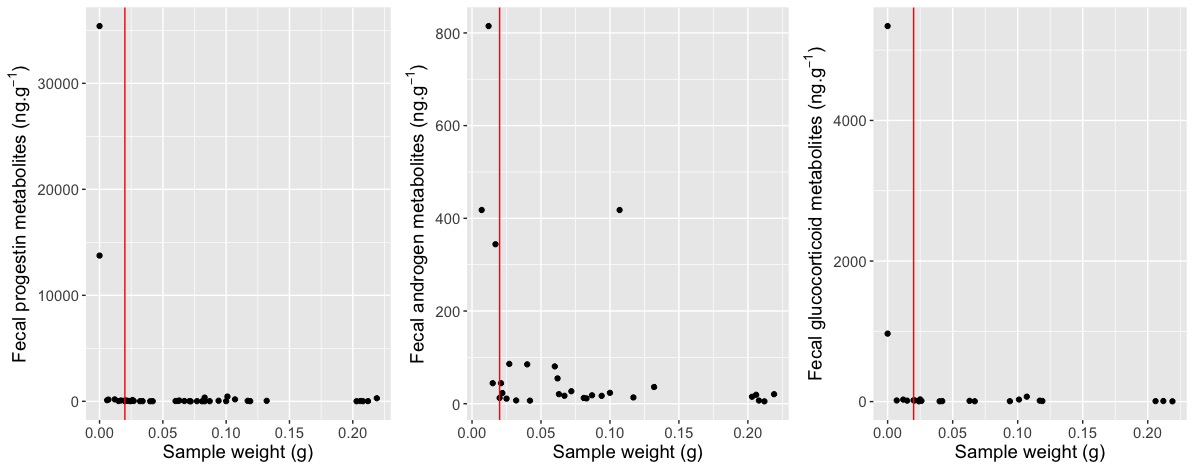


Figure S1: Relationship between sample weight (dried mass) and fecal hormone metabolite values from gray whale samples collected along the Oregon coast, USA. Vertical red lines represent the cut-off point of 0.02 g.


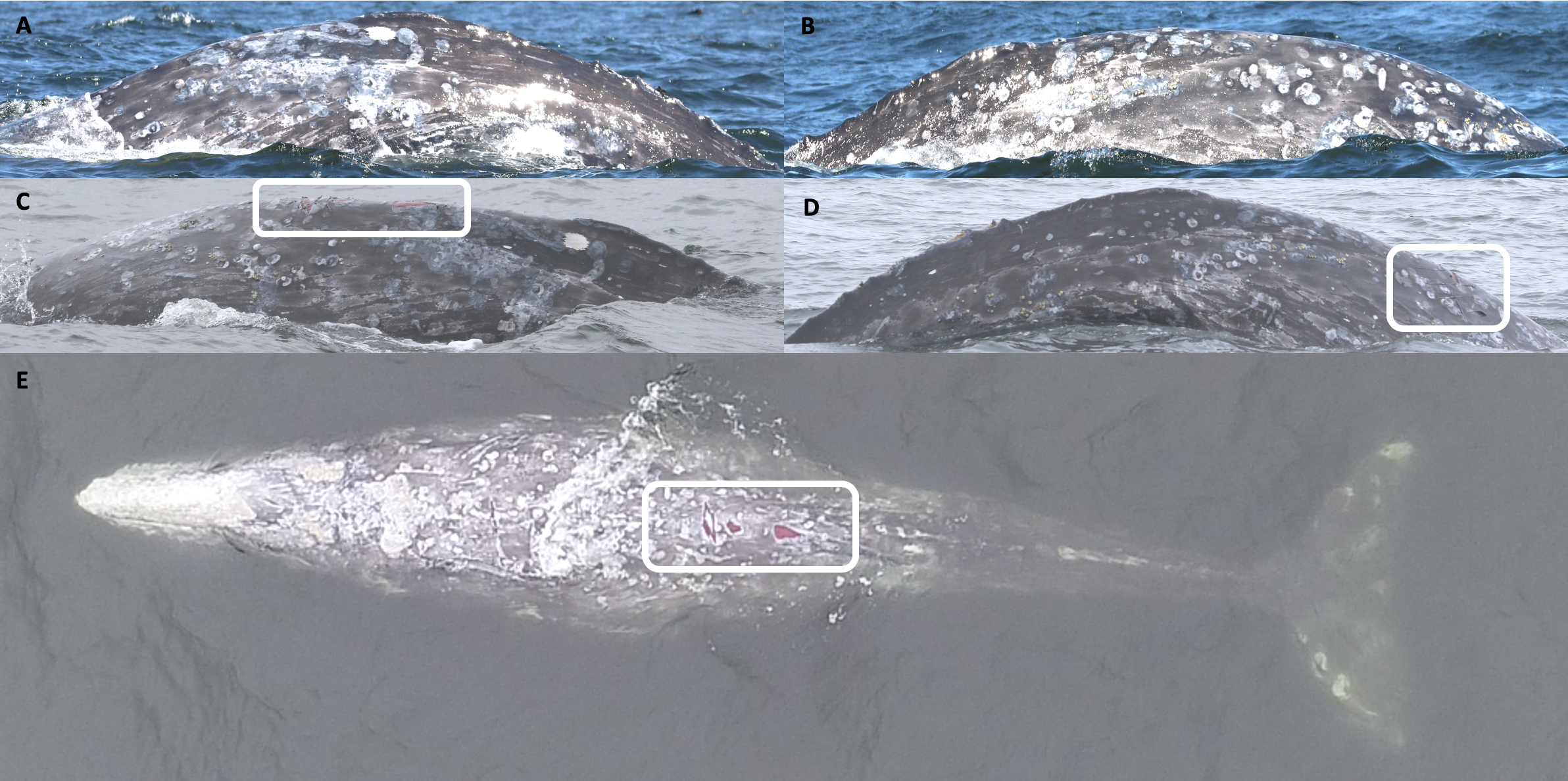


Figure S2: Case study of an immature male individual (whale ID: ErPNW-223) whose fecal sample was collected within 24 hours of a propeller/vessel strike injury and may reflect a typical endocrine profile of physiologically stressed individuals. Left (A) and right (B) flank sides of the whale on 19 June 2018 with no injuries; left (C) and right (D) flank sides of the whale on 20 June 2018 with injuries consistent with a propeller/vessel strike; (E) aerial drone view of the whale on 20 June 2018 with injuries. White rectangles highlight the injuries.


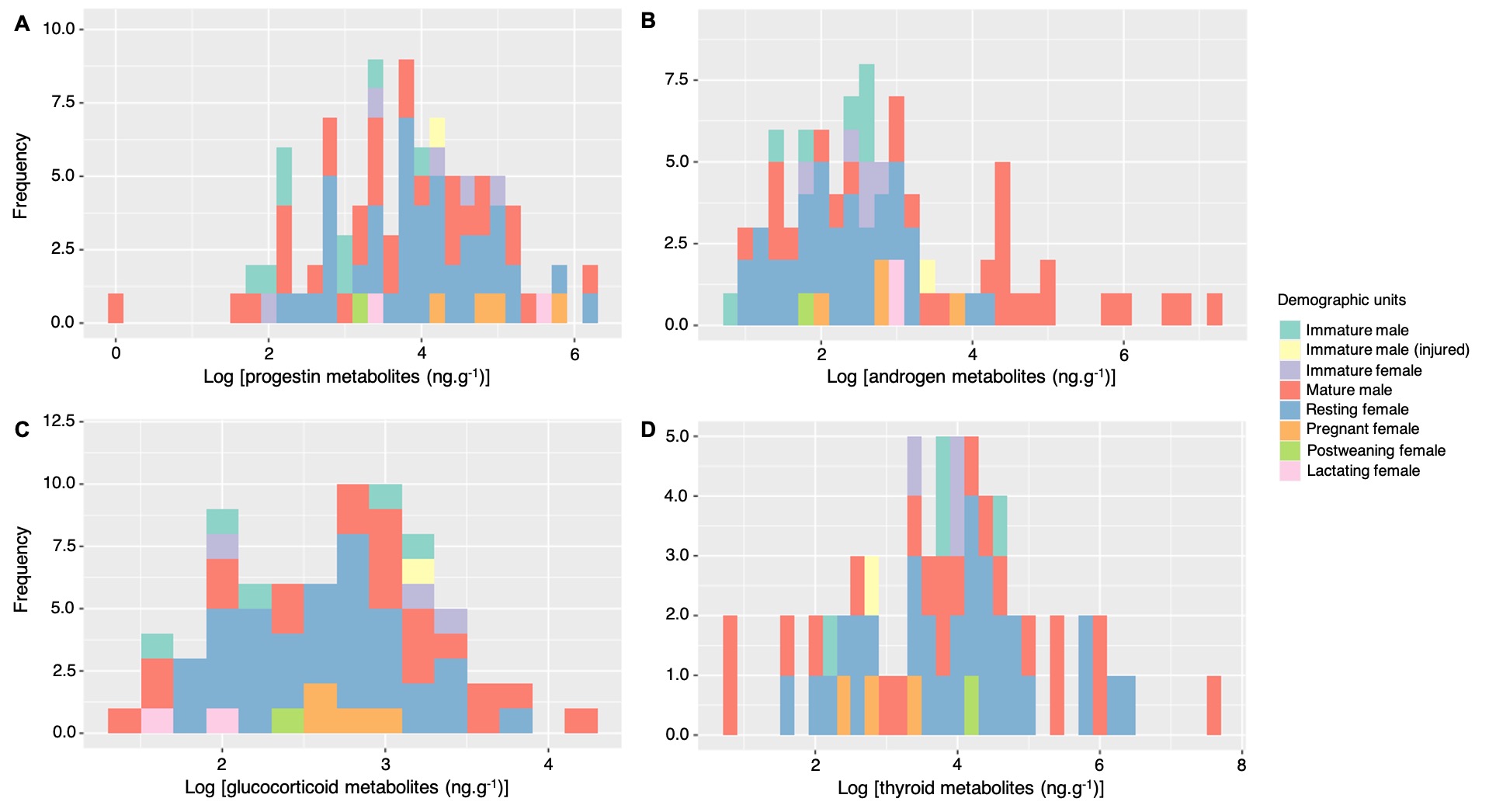


Figure S3: Frequency histograms of gray whale fecal hormone metabolite concentrations (dried mass) during June to October of 2016-2018 off the Oregon coast, USA. Individual whales may be represented multiple times in these plots as they may be re-sighted within and between years, and also being classified into various demographic units per year (i.e., lactating to postweaning females).

**References**

Arbor Assays, DetectX Triiodothyronine (T3) Enzyme Immunoassay Kit Species Independent. In: A. Assays, (Ed.). Arbor Assays, 2016.

Enzo, Progesterone ELISA kit. In: E. L. Sciences, (Ed.), Product Manual. Enzo Life Sciences, 2014.

Enzo, Cortisol ELISA kit In: E. L. Sciences, (Ed.), Product Manual. Enzo Life Sciences, 2015a.

Enzo, Testosterone ELISA kit. In: E. L. Sciences, (Ed.), Product Manual. Enzo Life Sciences, 2015b.
